# American Pharmaceutical Association

**Published:** 1892-01

**Authors:** 


MEMBERSHIP in the American Pharmaceutical Association is obtained only by election at the annual meeting. "Every pharmacist and druggist of good moral and professional standing, whether in business on his own account, retired from business, or employed by another, and those teachers of pharmacy, chemistry and botany who may be especially interested in pharmacy and materia medica" are eligible for membership. For blank application and further information, address Dr. H. M. Whelpley, 2729 Washington avenue, St. Louis, Mo., Chairman of the Committee on Membership.

